# Oligonucleotide Insecticides for Green Agriculture: Regulatory Role of Contact DNA in Plant–Insect Interactions

**DOI:** 10.3390/ijms232415681

**Published:** 2022-12-10

**Authors:** Volodymyr V. Oberemok, Refat Z. Useinov, Oleksii A. Skorokhod, Nikita V. Gal’chinsky, Ilya A. Novikov, Tatyana P. Makalish, Ekaterina V. Yatskova, Alexander K. Sharmagiy, Ilya O. Golovkin, Yuri I. Gninenko, Yelizaveta V. Puzanova, Oksana A. Andreeva, Edie E. Alieva, Emre Eken, Kateryna V. Laikova, Yuri V. Plugatar

**Affiliations:** 1 Department of Molecular Genetics and Biotechnologies, Institute of Biochemical Technologies, Ecology and Pharmacy, V.I. Vernadsky Crimean Federal University, Simferopol 295007, Crimea; 2 Laboratory of Entomology and Phytopathology, Dendrology and Landscape Architecture, Nikita Botanical Gardens—National Scientific Centre of the Russian Academy of Sciences, Yalta 298648, Crimea; 3 Department of Life Sciences and Systems Biology, University of Turin, 10124 Turin, Italy; 4 S.I. Georgievsky Medical Academy, V.I. Vernadsky Crimean Federal University, Simferopol 295015, Crimea; 5 All-Russian Research Institute for Silviculture and Mechanization of Forestry, Pushkino 141200, Russia; 6 Department of Natural Ecosystems, Nikita Botanical Garden—National Scientific Centre of the Russian Academy of Sciences, Yalta 298648, Crimea

**Keywords:** extracellular DNA, oligonucleotide insecticides, olinscides, DNA insecticides, 28S rRNA, gene expression, environmentally-friendly insect pest control

## Abstract

Insects vastly outnumber us in terms of species and total biomass, and are among the most efficient and voracious consumers of plants on the planet. As a result, to preserve crops, one of the primary tasks in agriculture has always been the need to control and reduce the number of insect pests. The current use of chemical insecticides leads to the accumulation of xenobiotics in ecosystems and a decreased number of species in those ecosystems, including insects. Sustainable development of human society is impossible without useful insects, so the control of insect pests must be effective and selective at the same time. In this article, we show for the first time a natural way to regulate the number of insect pests based on the use of extracellular double-stranded DNA secreted by the plant *Pittosporum tobira*. Using a principle similar to one found in nature, we show that the topical application of artificially synthesized short antisense oligonucleotide insecticides (olinscides, DNA insecticides) is an effective and selective way to control the insect *Coccus hesperidum*. Using contact oligonucleotide insecticide Coccus-11 at a concentration of 100 ng/μL on *C. hesperidum* larvae resulted in a mortality of 95.59 ± 1.63% within 12 days. Green oligonucleotide insecticides, created by nature and later discovered by humans, demonstrate a new method to control insect pests that is beneficial and safe for macromolecular insect pest management.

## 1. Introduction

The human population continues to grow, which requires expanding the food industry to meet the nutrient requirements of a growing society. Currently, the human population in biomass terms is approximately 0.06 gigatons of carbon. While there are now more of us than have ever existed on this planet, our numbers are dwarfed by those of insects, the vast majority of which comprise terrestrial arthropods, whose biomass is approximately 1.3 gigatons of carbon [1]. Insects are our main competitors when it comes to the crops humans depend on. In modern agriculture, losses to insect pests are 30% of pre-crop and 10% of post-crop yields [2,3], resulting in significant economic losses in both small and large industries. The estimated losses in crop yields are due mainly to insect pests [4].

Intensive agriculture involves using pesticides on a large scale, which is predicted to increase 2.7-fold by 2050 as the human population nears 9 billion [5]. Global use of highly effective chemical pesticides, on the one hand, leads to genetic resistance among insect pests [6,7], which creates the need for the never-ending development and production of new preparations. On the other hand, realizing the potential of agrocenosis means ensuring that they can reach and maintain a stable ecological balance. One important way to do this is through the creation and use of insecticides without upsetting or destroying the ecological balance.

Numerous experts believe that no serious alternative to chemical insecticides exists [8], as they are cheaper to manufacture and faster-acting than biological agents. For humans, time and money, which we can immediately and easily quantify, govern our actions more than difficult-to-assess future threats to water, air, land, and climate. Thus, there is a need to unite the best characteristics of biological and chemical preparations in a new generation of insecticides, which should be effective, affordable, and, most importantly, environmentally friendly. We cannot build a sustainable global human society if we fail to preserve natural biodiversity within a stable natural environment. If the use of modern chemical insecticides continues, people and the environment will be exposed to great dangers and the environmental risks [9,10,11] associated with the accumulation of xenobiotics in soil and groundwater [12], decreased numbers of pollinating insects [13], and negative effects on the health of humans, animals, and the plants on which both groups depend.

To solve this problem, the development of DNA insecticides (oligonucleotide insecticides, or olinscides) is a promising direction of research [2,14,15,16]. The concentrations of modern chemical insecticides (organic xenobiotics) in ecosystems could be reduced by using DNA insecticides, which act on and are metabolized by ubiquitous DNAses [15]. Using short antisense fragments from the conserved regions of the genes of insect pests opens the possibility of using this approach when resistance to conventional insecticides occurs. Oligonucleotide insecticides could be created by changing only the combination of nitrogenous bases depending on the gene sequences of the target insect pest. Our research suggests that antisense fragments of ribosomal genes are very promising for use as olinscides [14,15,16]. While mRNA comprises just 5% of cellular RNA, more abundant rRNA accounts for 80% of cellular RNA and is metabolically stable [17,18], making it an excellent candidate for silencing through antisense oligonucleotides. Data from our recent studies show that DNA insecticides designed for gypsy moth larvae can be selective and thus non-harmful both for non-target insects, such as black cutworm and tobacco hornworm [19], and for plants such as wheat [20], oaks, and apple trees [15,21]. The striking selectivity of the action of oligonucleotide insecticides, shown in our recent experiments, when replacing only one nitrogenous base at the 5′- and 3′-ends of olinscides and dramatically reducing their efficiency, reveals great opportunities for creating well-tailored insecticides, which potentially have little to compare with in modern plant protection (under publication).

An analog of DNA insecticides found in nature is extracellular DNA (exDNA). More and more studies show the presence of degraded DNA on the surface of plants and associate its presence with the performance of various functions, primarily protective [22,23,24]. The current knowledge suggests that the formation of a nanolayer of exDNA by plants on the surface of leaves, stems, and roots could be a vital adaptation developed during evolution to increase the general fitness of the particular species.

Recent trends in research on plants have provided evidence that exDNA is often actively secreted by plants and used to perform several tasks, thereby offering an attractive target or tool for biotechnological, medical, environmental, and general microbiological applications. The secreted exDNA results from either cell lysis or active release and can be found in both double- and single-stranded and more or less fragmented forms [24,25,26,27]. These DNA are ubiquitous and can be found on all types of environmental samples. The presence of exDNA in the growth medium of plants enhances the growth of lateral roots and root hairs, an effect linked to altered expression of the specific peptide hormone genes controlling root morphology [28]. In this context, exDNA functions as a signaling compound. Owing to its negative charge, exDNA acts as a chelator of cationic antimicrobials [29], though it can also provide protection against aminoglycosides [30] and activate the human innate immune system by interacting with pattern recognition receptors [31].

In the literature regarding extracellular DNA, examples of the insecticidal effect of DNA found on the surface of leaves are absent. It would be useful to gain a better understanding of their role in the extracellular environment, which seems to be to protect the plant from harmful external factors. Extracellular DNA is a kind of invisible “nanoshield” that, on the one hand, warns parasites, and on the other hand, maintains the boundaries that separate the individual plant from other individuals of the same species and individuals of closely related species [23,32]. In this article, we will, for the first time, investigate the insecticidal effect of exDNA of *Pittosporum tobira Thunb. (Apiales: Pittosporaceae*) and artificially synthesized short antisense oligonucleotide Coccus-11 on the viability of soft scale insect *Coccus hesperidum* L. (*Hemiptera: Coccoidae*), a cosmopolitan and polyphagous pest species [33,34] causing significant damage to citrus crops, mango, guava, and lychee [34]. *C. hesperidum* may have the capacity to affect approximately 125 plant families [35]. Outbreaks of scale insects may cause economic losses due to the direct feeding on cell nutrients and reduction of photosynthetic leaf area. Losses may affect fruit yields (e.g., citrus) or reduce the cosmetic value of ornamental plants. Damages caused to plants include the loss of sap and the clogging of leaf or fruit surfaces with honeydew, on which sooty mold subsequently grows [33,36].

## 2. Results

**Figure 1:**
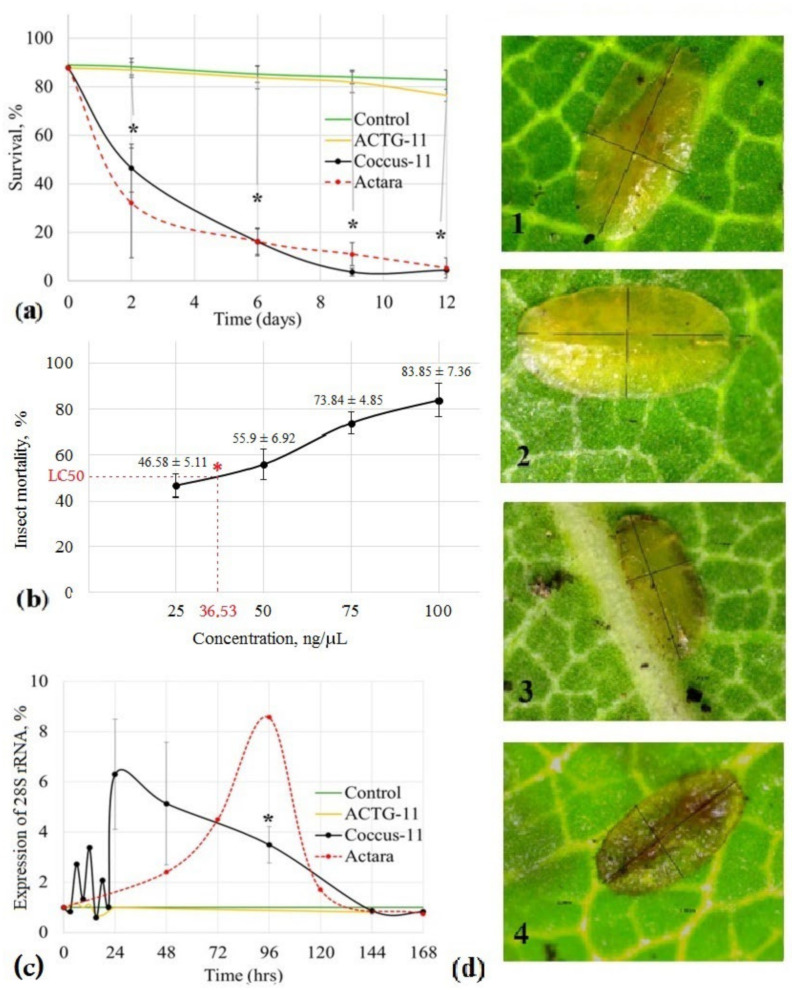
The effect of the ssDNA insecticide Coccus-11 on *C. hesperidum* larvae. The *C. hesperidum* larvae were treated topically with ssDNA insecticide (Coccus-11), with commercial neonicotinoid insecticide Actara^®^ (Actara), or with control ssDNA oligonucleotide ACTG-11 (ACTG-11) and water (Control). (**a**) Survival rate was calculated; (**b**) dose-effect curve for Coccus-11 (6th day of the experiment); LC50 is indicated by a red asterisk; (**c**) expression of 28S rRNA was analyzed by PCR, control was taken as 100%; (**d**) insect morphology was investigated with light microscopy: 1—control larva with intact integuments, 2—larva from ACTG-11 group with intact integuments, 3—larva from Coccus-11 group with tissue necrosis at the edges of larva body, 4—larva from Actara group with noticeable darkening of the body. Means and standard error of means are represented on panels (**a**,**b**); * is marked when *p* < 0.01.

We evaluated the effect of a short 11-mer antisense oligonucleotide insecticide Coccus-11 (single-stranded DNA, ssDNA) at a concentration of 100 ng/μL on the viability of *C. hesperidum* larvae and found significant mortality as early as the 2nd day (53.53% ± 9.99%; *p* < 0.01) (Figure 1). Similar results were obtained on the 2nd day using the neonicotinoid insecticide Actara^®^ at a concentration of 0.8 g/L (68.94 ± 22.63%; *p* < 0.01) (Figure 1). Mortality progressively increased in the Coccus-11 group up to the 9th day. By the end of the experiment on the 12th day, mortality reached 95.59 ± 1.63% (*p* < 0.01). Similar data were obtained for Actara^®^. By the end of the experiment in the Coccus-11 group, there was a mortality of 94.7 ± 4.11% (*p* < 0.01), confirming that Coccus-11 DNA insecticide is comparable in efficacy to Actara^®^. The control oligonucleotide ACTG-11 did not show any significant insecticidal effect compared with the Control group (treated with water). The mortality rate in the ACTG-11 group was 23.53 ± 2.51% (*p* = 0.14) on the 12th day (Figure 1a). In the water-treated Control group, a distinct factor existed that led to a low mortality rate that remained at 10–15% over the year (our observations in 2019 and 2020). We also created a dose-effect curve and found that LC50 for *C. hesperidum* larvae was 36.53 ng/μL (Figure 1b).

To evaluate any possible negative effects of Coccus-11 DNA oligonucleotide on the plant, we measured the pH of the leaves, which was 6.02 ± 0.05 in the Coccus-11 group and 6.08 ± 0.01 in the Control group (*p* > 0.05). No significant difference was found, confirming the environmental safety of Coccus-11 DNA insecticide. Encouraged by the results of the insecticide activity, we evaluated the specificity of action of the DNA insecticide Coccus-11 by studying the target 28S rRNA. A decrease in the expression of the target gene is the gold standard for proof of specificity of action [37] for antisense oligonucleotides. Surprisingly, in this study, we found an increase in the expression of the target 28S rRNA.

Expression increased in a sinusoidal manner in response to the application of the DNA insecticide Coccus-11 and then gradually underwent a smooth decrease. The maximum increase in the expression of 28S rRNA was detected at 24 h and was more than 6-fold higher than in the Control group (6.31 ± 2.44; Figure 1c). The significant difference compared to the Control group was observed on the 4th day (3.49 ± 0.72, *p* < 0.01), corresponding to the period of massive death of treated insects. A possible explanation for this cell reaction is the overcompensation of the gene suppression since 28S rRNA and other rRNAs are vital for protein biosynthesis [38]. Coccus-11 altered ribosome biogenesis, which is tightly linked to the control of cellular growth and proliferation. When we inspected the appearance of the insects, we found blackening of the body edges as a result of cell death in the Coccus-11 (Figure 1) group and blackening of the entire insect in the Actara^®^ group (Figure 1, panel d4), while the Control group and ACTG-11 group had no visible changes (Figure 1, panel d1, d2). Actara^®^ also significantly increased the expression of 28S rRNA on the 4th day (8.57 ± 0.36, *p* < 0.01). Apparently, insect tissues try to substitute for dead cells, which eventually leads to an increase in the expression of 28S rRNA, cell depletion, and the death of the whole insect. Previously, we also recorded an increase in the expression of ribosomal genes in response to antisense oligonucleotides in insect pests: *Dynaspidiotus britannicus* Comstock, *Unaspis euonymi* Newstead, and *Ceroplastes japonicus* Green (unpublished data).

**Figure 2:**
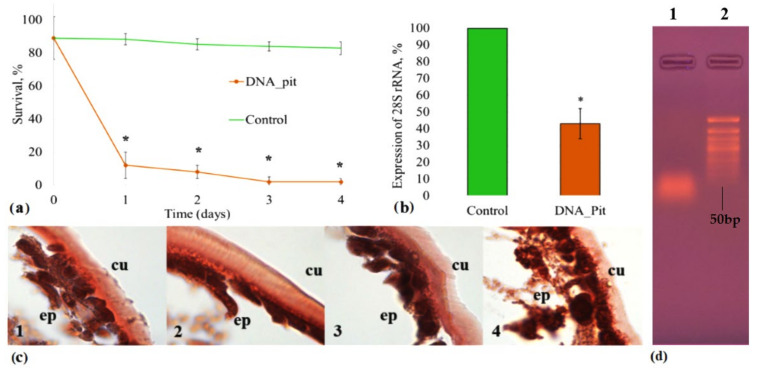
The effect of the plant’s exDNA on *C. hesperidum* larvae. The *C. hesperidum* larvae were treated topically with P. tobira exDNA (DNA_pit) or with water (Control). (**a**) Survival rate was calculated; (**b**) expression of 28S rRNA was analyzed by PCR (Control is taken as 100%); (**c**) insect histology (using Congo red) was investigated with light microscopy: 1—Control; 2—ACTG-11; 3—Coccus-11; 4—DNA_pit (explanation in the text); (**d**) electrophoregram (1.8% agarose gel): 1—exDNA of *P. tobira* leaf surfaces, 2—DNA ladder 50 bp. Means and standard error of means are represented on panels (**a**,**b**); * is marked when *p* < 0.01.

We then undertook the task of detecting a similar insecticidal effect in nature. Our attention was attracted by exDNA on the surface of *P. tobira* since we assumed that the death of the insects in the water-treated Control group (10.01 ± 2.08%) could be related to the degraded plant extracellular DNA presented on the plant epidermis. DNA sequencing showed that the degraded DNA on the leaf surfaces of *P. tobira* belonged to the plant itself, and electrophoresis showed that the length of this exDNA was predominantly around 50 bp (Figure 2b). We concentrated this DNA to 100 ng/μL, purified it with HPLC, and used it to treat the *C. hesperidum* insect larvae (DNA pit group in Figure 2d).

In nature, the concentration of extracellular DNA on *P. tobira* leaves was 24.371 ± 0.971 ng per cm^2^ of the leaf. We assumed that this DNA is a regulator of the number of insect pests. To test this hypothesis, we evaluated the effect of total concentrated exDNA (100 ng/μL) on the viability of *C. hesperidum* larvae. We found a highly significant mortality rate of 89.47 ± 7.3% (*p* < 0.01) on the 1st day and 98.14 ± 2.5% (*p* < 0.01) on the 4th day in the DNA_pit group. Interestingly, when an exDNA concentration of 10 ng/μL was used for treatments, insect mortality dropped to 9.8 ± 2.3% on the 4th day. Mortality in the Control group was was not more than 17.05 ± 3.8% throughout the experiment (Figure 2a). It is obvious that the death of the insects, in this case, occurred due to the complex effect (probably acting on many analogous genes in the insect) of the degraded plant’s exDNA. Nevertheless, the strong decrease in the expression of 28S rRNA in response to the use of degraded exDNA (2.48 ± 0.52; *p* < 0.01) was detected on the 4th day in comparison with the Control group (Figure 2b).

Upon histological examination with Congo red [39], we found that in the Control group, the insect epithelial cells (the first barrier that meets DNA insecticide molecules) contained small brick-red granules, and the cuticle had two layers, of which the inner layer was more intensely colored (Figure 2, panel c1). Interestingly, in the ACTG-11 group, the color saturation of the cuticle was the same as that in the Control group, and the ratio of layer thicknesses was similar (Figure 2, panel c2). In the Coccus-11 group, an accumulation of protein granules in the cytoplasm was observed, with a decrease in the color saturation of the entire cuticle and its thickness (Figure 2, panel c3). Such changes in the Coccus-11 group may indicate a weak protein excretion by epithelial cells connected with altered protein biosynthesis. In the DNA_pit group, the epithelial cells had the brightest color among all groups and contained the largest number of protein granules. Protein export from the cell was discontinued, and the cuticle had only one outer, faintly colored layer (Figure 2, panel c4).

We also observed that at the macro-level, in the ssDNA and exDNA treated groups (Coccus-11 and DNA_pit groups), it was easier to separate the cuticle from the insect body, which was a damaging consequence of a violation of the cuticle architecture. Additionally, we noted the long-term ssDNA treatment effect after one year of periodical *P. tobira* plant treatment with Coccus-11 ssDNA, and we observed a 7-fold (7.05 ± 1.91; *p* < 0.05) decrease in the occurrence of *C. hesperidum* per plant in comparison with the water- treated Control group. It is obvious that the exDNA of *P. tobira* in low concentrations in natural conditions can slightly reduce the survival rate of the insect and may be the cause of the small mortality (10.01 ± 2.08%) of the insects found in the water-treated Control group (Figure 2a; Control group line).

## 3. Discussion

Recently, scientists have been actively studying how an organism’s exDNA influences its growth and development. However, little attention has been paid to its participation in interspecies interactions. In our study, we tried to answer the question of whether the DNA of one organism can regulate the number of other organisms and whether this mechanism could be used in plant protection. The key task of the study, which began in 2008 by Oberemok’s group [38], was the search for natural DNA insecticides and the development of complete insecticides by in vitro synthesized oligonucleotides.

In this article, we show for the first time the protective function of degraded double-stranded exDNA on the leaf surface of *P. tobira*, which causes the death of *C. hesperidum* larvae. We found that in nature, the leaf-covering plant exDNA is around 50 bp in length and has insecticidal properties, as we show in this study. Of note, length of *P. tobira* exDNA is shorter in summer than in winter and can be explained by stronger UV radiation that degrades DNA. We believe that this degraded exDNA acts by a mechanism similar to DNA interference [40,41] and can comprehensively affect the work of many systems or genes inside an insect pest cell since insect pests and host plants may possess similarities in genome sequence [42]. The concentration of the degraded DNA found on the leaf surface to 100 ng/μL led to almost 100% death of the insect pests. From this, it can be assumed that the plant itself can reduce the number of insect pests on its leaf surfaces. Therefore, the presence of exDNA could contribute to the mortality of insects in the Control group, which usually occurred in the range from 9% to 20%. It can be assumed that many plants can use their degraded DNA to protect themselves from sucking insects, an evolutionary adaptation that allows them to fine-tune the parasite-host relationship. The data also reveal the potential use of genomic DNA as a tool for influencing not only a plant’s own cells but also other organisms interacting with the plant, an important finding for ecology.

On the other hand, in our study, we found that synthetic antisense oligonucleotides, particularly Coccus-11, have an insecticidal effect and are analogous to the degraded exDNA secreted by the plant. The difference is that the antisense oligonucleotide specifically up-regulates the expression of 28S rRNA (as Actara^®^ does) while the plant’s exDNA down-regulates it. It is a surprising result for antisense oligonucleotide Coccus-11 and requires further investigation. At the same time, a random ACTG-11 fragment did not show any significant effect. These data provide excellent evidence for the creation of selectively-acting oligonucleotide insecticides. Gene expression detected in this study differed from the typical effect seen when using antisense oligonucleotides. Instead of suppressing the expression of a target gene, a wave-like dynamics of an increase in gene expression and then a smooth decrease was found, and may be a characteristic pattern of 28S rRNA gene silencing in insects.

**Figure 3:**
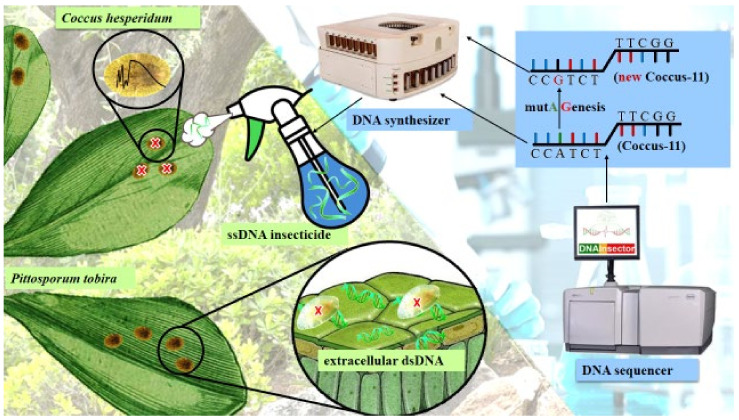
The concept of green DNA insecticides for nature.

Today, DNA insecticides (oligonucleotide insecticides, briefly olinscides) as oligonucleotide biological treatment can compete with modern chemicals. They have a high potential for use in producing safe and effective insecticides targeting hemipteran insects, which has also been demonstrated in our recent work for *D. britannicus* and *C. japonicus* [14,16]. As the example of Coccus-11 demonstrates, it is possible to develop DNA insecticides for a wide range of insects adopting this strategy, illustrated in Figure 3. In this regard, oligonucleotide insecticides have a great advantage: in addition to their selective effect on the target insect, such preparations can now be produced quickly and in large quantities. Even if genetic mutation resistance should be developed, the preparations can be quickly modified using another sequence to return the DNA insecticides to their proper effectiveness.

## 4. Materials and Methods

### 4.1. Origin of C. hesperidum L.

We identified *C. hesperidum* larvae (*Hemiptera: Coccidae*) on *P. tobira* Thunb. (*Apiales: Pittosporaceae*) in the Nikita Botanical Garden (Yalta, Crimea), collected them and used them for our experiments in 2019–2021.

### 4.2. Applied DNA Sequences

#### 4.2.1. ssDNA Coccus-11

We used one algorithm of web application, DNAInsector (dnainsector.com), to choose the Coccus-11 (5′–CCA–TCT–TTC–GG–3′; GenBank: MT317022.1) sequence as a contact ssDNA insecticide dissolved in nuclease-free water (100 ng/μL) applied, using a hand sprayer, to *P. tobira* leaves (1 mg of ssDNA insecticides per m^2^ of leaves). As a control ssDNA fragment, we used ACTG-11 (5′–ACT–GAC–TGA–CT–3′) in the same concentration. All sequences were synthesized using an ASM 800E DNA synthesizer (Biosset, Russia). An additional control water-treated group was used (without single-stranded oligonucleotides). During 8 repeated experiments, around 1000 larvae were treated, which were included in the statistical sample to assess the survival rate. Insects were counted for each replication of each variant of the experiment on 20 leaves of *P. tobira*. Mortality was calculated by dividing the number of dead individuals by the total number of individuals on the leaf and multiplying by 100%. Insects were counted using a Nikon SMZ745 microscope and a Toupcam ucmos 5100 kpa camera to take photos of insects.

#### 4.2.2. dsDNA from *P. tobira* (DNA_pit)

DNA was collected in summer from the leaf surfaces of *P. tobira* with wet cotton balls and filtered on a 0.2-micron syringe filter (Whatman, Maidstone, UK; Dassel, Germany). Then the pure filtrate was evaporated to dryness under a vacuum (Heidolph, Germany). The separation of fractions was carried out on an HPLC (Knauer, Germany). Separate fractions were collected in Eppendorf tubes and evaporated on a Concentrator plus (Eppendorf, Hamburg, Germany) under a vacuum to dryness; then, the dry residue was diluted with Milli-Q grade water to 100 ng/μL. The electrophoresis of the nucleic acids on agarose gel demonstrated that the length of the main fraction of the DNA was around 50 bp.

### 4.3. HPLC Assay

The analysis and purification of oligonucleotides by reverse-phase high-performance liquid chromatography (RP-HPLC) was performed on a Jupiter 5 µm C18 300 Ǻ (4.6 mm × 250 mm) column using a preparative HPLC system (Azura P6.1 L, UVD 2.1S detector). Buffers used for analysis were (A) 0.1 M triethylammonium acetate in water and (B) 50% MeCN/buffer A. The gradient was 30%, and the flow was 1 mL/min.

### 4.4. Neonicotinoid Insecticide Treatment

To assess the effectiveness of the action of the DNA insecticides, the *C. hesperidum*-approved chemical neonicotinoid insecticide Actara^®^ (0.8 g/L) was used as a standard.

### 4.5. Evaluation of 28S rRNA Expression of C. hesperidum

*C. hesperidum* larvae were ground using a pestle in a 1.5 mL tube to prepare them for RNA extraction by ExtractRNA kit (Evrogen, Russia) according to the manufacturer’s instructions. Three independent extractions were carried out to produce the replicates for each treatment. For each extraction, 10 larvae were used from each group (Control, ACTG-11, Coccus-11, DNA_pit). The quality and concentration of the extracted total RNA were assessed using a NanoDrop spectrophotometer (Thermo Scientific, Waltham, MA, USA). 1.5% agarose gel was used to run electrophoresis in TBE (Tris-borate-EDTA) buffer (10 V/cm) for 30 min, and 5 µL of the eluted RNA volume was loaded for each group. Among all experimental groups, the quantity, intensity, and pattern of the RNA bands were equal.

For reverse transcription, the total RNA (50 ng) was annealed with Coccus-R primer (5′–ACG–TCA–GAA–TCG–CTG–C–3′) and analyzed using a FastStart Essential DNA Green Master (Roche, Germany), according to the manufacturer’s instructions. The reaction was conducted at 40 °C for 60 min in a LightCycler^®^ 96 (Roche, Basel, Switzerland). For each sample, a 0.5 µL aliquot of the obtained cDNA was used for each PCR reaction. Primers, forward 5′–ACC–GTC–GAC–GAA–CTG–G–3′ and reverse 5′–ACG–TCA–GAA–TCG–CTG–C–3′, were used for quantitative real-time PCR studies and amplification with gene-specific primers to quantify the *C. hesperidum* 28S rRNA. The qPCRmix-HS SYBR (Evrogen, Russia) master mix was used according to the manufacturer’s instructions. The following procedure of 10 min initial denaturation at 95 °C, followed by 30 cycles with 10 s denaturation at 95 °C, 15 s annealing at 62 °C, and 14 s elongation at 72 °C was used for amplification on a LightCycler^®^ 96 instrument (Roche, Basel, Switzerland). PCR was repeated in triplicate for each condition. Finally, to estimate the specificity of amplification and the presence of additional products, all the PCR products were melted.

### 4.6. Statistical Analyses

For statistical analysis, the standard deviation (SD) and the standard error of the mean (SE) were determined and evaluated using the Student’s *t*-test; *p* < 0.01 was considered significant (Microsoft Excel software, Redmond, Washington, DC, USA).

## 5. Conclusions

In summation, it should be noted that all biological components of ecosystems (producers, consumers, and reducers) are interconnected via many ecological links. The chemical insecticides in widespread global use are difficult to control. As they become involved in trophic chains, they become more mobile. As they move away from the areas to which they were first applied, they can collect and concentrate. In addition, they can undergo biotransformation and increase in toxicity. Penetration of pesticides into deep soil layers leads to groundwater pollution. Since most modern chemical insecticides have a relatively long half-life, an increase in the concentration of the target chemical agent in the ecosystem occurs during the transition from a low to a higher trophic level. As a result of the use of persistent chemical insecticides in agriculture and forestry, they will always poison the participants in the trophic levels of aquatic and terrestrial ecosystems, where for most xenobiotics there are no enzymes that can catalyze their rapid decomposition. Thus, the only safe way to control the number of insect pests is to use natural molecules that can be safe and effective simultaneously. At the moment, it is known that only nucleic acids, RNA, and DNA, with certain sequences, can selectively control a wide spectrum of metabolism activities in the cells of target insect pests.

The use of short antisense fragments from the conserved parts of the ribosomal genes of insect pests opens up the possibility of using them in anti-resistance programs, as well as creating green DNA insecticides by a universal mechanism, changing only the sequences of oligonucleotide insecticide depending on the gene sequences of the target insect pest. Thus, the regulatory role of DNA goes beyond the cell nucleus and cells itself and can be an effective tool for controlling the number of insect pests prompted by nature.

## Data Availability

Not applicable.
